# Peptide Receptor Radionuclide Therapy and Primary Brain Tumors: An Overview

**DOI:** 10.3390/ph14090872

**Published:** 2021-08-29

**Authors:** Andrea Cimini, Maria Ricci, Francesca Russo, Martina Egidi, Ferdinando Calabria, Antonio Bagnato, Orazio Schillaci, Agostino Chiaravalloti

**Affiliations:** 1 Department of Biomedicine and Prevention, University Tor Vergata, 00133 Rome, Italy; maria.ricci28@gmail.com (M.R.); francescarusso.91@outlook.it (F.R.); smartix88@gmail.com (M.E.); orazio.schillaci@uniroma2.it (O.S.); agostino.chiaravalloti@gmail.com (A.C.); 2 Department of Nuclear Medicine and Theragnostics, “Mariano Santo” Hospital, 87100 Cosenza, Italy; ferdinandocalabria@hotmail.it (F.C.); alfabag@gmail.com (A.B.); 3 Department of Nuclear Medicine, IRCCS Neuromed, 86077 Pozzilli, Italy

**Keywords:** radiopharmaceuticals, peptide receptor radionuclide therapy, primary brain tumors, theragnostics, nuclear medicine

## Abstract

Primary brain tumors (PBTs) are some of the most difficult types of cancer to treat, and despite advancements in surgery, chemotherapy and radiotherapy, new strategies for the treatment of PBTs are needed, especially for those with poor prognosis such as inoperable/difficult-to-reach lesions or relapsing disease. In regard to the last point, malignant primary brain tumors remain some of the most lethal types of cancer. Nuclear medicine may provide exciting new weapons and significant contributions in the treatment of PBTs. In this review, we performed literature research in order to highlight the possible role of peptide receptor radionuclide therapy (PRRT) in the treatment of PBTs with radiolabeled molecules that bind with high-affinity transmembrane receptors such as somatostatin receptors (SSTRs), neurokinin type-1 receptor and prostate-specific membrane antigen (PSMA). These receptors are overexpressed in some cancer types such as gliomas, meningiomas, pituitary tumors and medulloblastomas. A comprehensive overview of possible applications in this field will be shown, providing knowledge about benefits, feasibility, developments and limitations of PRRT in this type of tumor, also revealing new advantages in the management of the disease.

## 1. Introduction

Primary brain tumors (PBTs) make up about 2% of all cancers, and they may be divided into benign and malignant [1,2]. In 2007, the World Health Organization (WHO) classified PBTs based on their histological aggressiveness with a grading scheme (from I to IV). In 2016, this classification was revised and updated, adding molecular alterations of PBTs. Gliomas represent 75% of malignant gliomas [3], and glioblastoma multiforme is the most lethal form. In fact, the prognosis of patients affected by glioblastoma multiforme remains a death sentence, with a median survival of 10–15 months [4,5,6]. 

The treatment of PBTs includes surgery, chemotherapy and radiotherapy, often combined [7]. It is well known that treatment of PBTs may be complicated due to multiple factors such as the blood–brain barrier (which prevents or slows down the arrival of chemotherapy drugs to the tumor mass) and the nature of the brain parenchyma itself, the removal of which (of the perilesional healthy tissue together with the tumor) must be limited as much as possible [8]. In addition, some lesions are difficult to reach or inoperable. Therefore, currently, the prognosis in these cases remains poor, with a survival of a few years. [9]. Taking into consideration the aforementioned context, and despite recent advances in surgery, radiotherapy and chemotherapy, new strategies for the treatment of PBTs are strongly needed. New possibilities to conventional therapies are represented by groundbreaking developments in radioligand therapy (RLT) in recent years, overcoming some of these challenges in PBTs [8]. RLT exerts its effect when the radiopharmaceutical binds to the cell surface, inducing DNA damage through radioactive decay. In this way, it is possible to hit more than one cancer cell per decay event (crossfire effect). It is also possible to couple the radionuclide to a selective targeting portion to target and kill cancer cells that express specific receptors, increasing the effectiveness of killing cancer cells without increasing off-target toxicity [1]. Several compounds may be marked with radionuclides, with chemical, immunological or molecular targets [10,11]. Radioisotopes that emit beta minus (β^−^) or alpha (α) particles are suitable for therapy. β^−^ particles can travel through tissues for a few millimeters but have a relatively low linear energy transfer (LET). The low LET can induce irreparable breaks of the DNA strand or more frequently repairable, thus causing mainly sublethal damage. β^−^-radiation can also cause cell killing indirectly, in an oxygen-rich environment, through the formation of reactive oxygen species (ROS). Both characteristics make the β^−^-particles more suitable for the treatment of large lesions, such as bulky and heterogeneous tumors. The use of α-particles, on the other hand, is more suitable for the treatment of small lesions (small volume tumors or clusters of tumor cells) due to their short travel distance in the tissue and high LET [12]. A further advantage of RLT is the use of radiopharmaceuticals as single-photon emission computerized tomography (SPECT)—or positron emission tomography (PET)—radiotracers by interchanging the cytotoxic radionuclide with a gamma (γ)- or positron (β+)-emitting radionuclide, respectively (in PET, γ rays that come from β+ decay are detected), therefore predicting the behavior of radioactive or nonradioactive targeted drugs (i.e., radiopharmaceuticals or pharmaceuticals) and allowing dosimetry analysis as well [1]. Currently, the term “theragnostics” indicates the use of the same targeting moiety for both diagnostics and therapy [13].

It is important to highlight the methods to deliver these therapeutic radiopharmaceuticals to PBT cells. Currently, strategies are represented by systemic delivery, intratumoral bolus injection, bolus injection into the tumor resection cavity and convection-enhanced delivery (CED), in which the radiopharmaceutical is infused directly into the tumor (circumventing the blood–brain barrier) [14,15]. Another promising strategy may be represented by intraarterial administration of the radiopharmaceutical. This technique may be useful to increase tumor uptake of the tracer, especially in cases in which tumor cells express low levels of target receptors [16].

In the field of RLT in PBTs, peptide receptor radionuclide therapy (PRRT) is gaining growing interest. In fact, in comparison to proteins, peptides have quicker diffusion and clearance due to their smaller size. In addition, peptides may be easily radiolabeled. For these characteristics, radiolabeled peptides are optimal probes for molecular imaging and RLT [17]. These radiopeptides bind with a high-affinity group of transmembrane proteins called G-protein-coupled receptors [17]. As regards PRRT in PBTs, possible applications are presented in Table 1, and the characteristics of radioisotopes used for therapeutic purposes are presented in Table 2.

The aim of this review is to provide a comprehensive overview of the most used radiopeptides used in PBTs for therapeutic purposes, also underlining theragnostic applications and highlighting possible advantages and drawbacks. We will report the most interesting studies regarding radiolabeled somatostatin analogs and radiopharmaceuticals for neurokinin type-1 receptors used for therapy of PBTs. Moreover, we will highlight possible applications in this field of radiopharmaceuticals for prostate-specific membrane antigen (PSMA) and possible future approaches. In addition, the safety and feasibility of PRRT in PBTs will also be discussed in this review.

## 2. Somatostatin Analogs and Somatostatin Receptors

Because several tumors express somatostatin receptors (SSTRs), targeted therapy with somatostatin analogs may be used for malignancy treatment. PBTs may present SSTRs as well, therefore allowing SPECT or positron emission tomography/computed tomography (PET/CT) imaging with somatostatin analogs and PRRT, which permits systemic treatment of cancer types that overexpress the transmembrane receptor [1,47,48]. 

Somatostatin is a peptide produced by the hypothalamus, and it acts as a neurohormone and neurotransmitter. A high variety of physiological functions are regulated by this hormone; furthermore, it presents inhibitory effects on the secretion of other hormones. The function of somatostatin is mediated by SSTR, a type of G-protein-coupled receptor [49]. These receptors are physiologically expressed in healthy tissues and organs such as the central and peripheral nervous system, pancreas and gastrointestinal tract, salivary glands and vessels, stimulating cell proliferation as well. SSTR are also expressed in inflammatory cells (in particular monocytes, lymphocytes and macrophages) [49]. After the interaction between somatostatin and SSTR, the complex ligand receptor is internalized in the cell [50].

There are five different subtypes of SSTR (SSTR1 to SSTR5). It is well known that SSTRs, especially subtype 2 (SSTR2), are overexpressed in different cancer types, especially in tumors of neuroendocrine origin (NET) [51,52]. In these cases, SSTRs are targeted with high affinity using somatostatin analogs, also allowing a theragnostic approach centered on radiolabeled somatostatin analogs using radioisotopes such as ^177^Lu, ^111^In, ^68^Ga and ^90^Y [53,54]. 

This approach utilizes an octreotide derivative as a targeting portion that is linked to a radioisotope using chelators such as tetraazacyclododecane-tetraacetic acid (DOTA) and diethylenetriamine pentaacetic acid (DTPA). In clinical practice, the two most used radiopeptides for therapeutic purposes are ^90^Y-DOTATOC ([[DOTA]^0-D-PHE1, [Tyr]^3] octreotide) and ^177^Lu-DOTATATE ([[DOTA]^0-[Tyr]^3] octreotate) [53,55,56]. On the other hand, gamma-emitting isotopes such as ^68^Ga or ^111^In may be used in somatostatin receptor imaging (SRI) in PET and SPECT, respectively, thus predicting the behavior of therapeutic radiopharmaceuticals and allowing biodistribution assessments as well. It is important to underline that DOTATOC presents high affinity for SSTR2 and low affinity for SSTR3 and SSTR5, whereas DOTATATE only binds to SSTR2 [57,58]. Consequently, the main target of PRRT with radiolabeled somatostatin analogs is represented by SSTR2. Nephrotoxicity is the most important limitation of PRRT with radiolabeled somatostatin analogs due to their renal excretion. Infusion of amino acids may be useful, reducing the renal uptake of radiopharmaceuticals [59,60]. Other side effects are represented by transient hematotoxicities such as anemia or leukopenia [35].

SSTR2 may be expressed by PBTs in children and adults. An interesting study by Lange et al. evaluated SSTR subtype expression in 57 patients with gliomas (astrocytic brain tumors, WHO grade from I to IV, age of the patients ranged from 1 to 81). SSTR2 was present in 25% of cases. Grade I and IV tumors showed a low expression of SSTR2, whereas a gradually increased SSTR2 expression was found from grade II to grade III tumors [19]. Contrasting results have been demonstrated in previous studies, in which SSTR2 overexpression in glioblastomas and low SSTR2 expression levels in WHO-grade-II–III gliomas were detected [20,21]. The potential role of PRRT in patients with gliomas that express SSTRs (and particularly SSTR2) has been demonstrated. In 2010, Heute et al. reported the treatment of three adult patients affected by recurrent glioblastoma multiforme with ^90^Y-DOTATOC. ^68^Ga-DOTATOC PET was performed prior to administration of the therapeutic radiopharmaceutical in order to assess the expression of SSTR2 in these tumors. Final results showed that PRRT was successful in all patients (one patient had complete remission in MRI and PET scans, and the other two showed partial remission), with no significant side effects reported. Moreover, patients showed an important improvement in their quality of life [24]. 

In an interesting study by Schumacher and collaborators, 10 adult patients affected by glioma (WHO grades II and III, 5 patients with progressive disease, 5 with debulked gliomas) were treated with ^90^Y-DOTATOC. It is important to highlight that in patients with progressive disease, tumor progression was halted for 13–45 months, and one inoperable anaplastic astrocytoma regressed into an operable lesion 2 years after therapy with ^90^Y-DOTATOC. In addition, the authors demonstrated the feasibility of PRRT following debulking. In all patients, PRRT was well tolerated, with no relevant side effects [25].

Pituitary tumors and meningiomas may express SSTRs, thus permitting a possible PRRT in some cases. As concerns pituitary tumors, SSTR subtypes are heterogeneously expressed [39]:

- Growth hormone (GH)-secreting tumors and thyroid-stimulating hormone (TSH)-secreting tumors mainly express SSTR2 and SSTR5 (SSTR2 is expressed in more than 90% of GH- and TSH-secreting tumors).
- SSTR1 and SSTR5 are the most represented subtypes in prolactinomas.
- Nonfunctioning and gonadotroph tumors mainly express SSTR3 (with lower levels of SSTR2 and SSTR1).
- SSTR5 is the most expressed subtype in adrenocorticotropic (ACTH)-secreting tumors.

Only a few reports have suggested the possible applications of PRRT in this type of tumor, and further studies on larger samples are required to assess the efficacy. Due to the heterogeneous distribution of SSTR in pituitary tumors, it is important to underline that somatostatin receptor imaging has a potential central role in the selection of potential patients for PRRT with radiolabeled somatostatin analogs [42]. In 2013, Komor et al. reported a case of patients with a relapsing macroadenoma with palsy of the oculomotorius nerve. After positive scintigraphy with ^111^In-pentetreotide that revealed the expression of SSTR2 in the lesion, the patient underwent therapy with ^177^Lu-DOTATOC. In this case, PRRT with a radiolabeled somatostatin analog was safe, with an improvement in ocular complications and tumor control for 8 years after PRRT [40]. In the same year, Maclean and collaborators reported a case of a patient with metastatic pituitary carcinoma that responded to PRRT with ^177^Lu-DOTATATE with stable disease [41]. 

**Figure 1:**
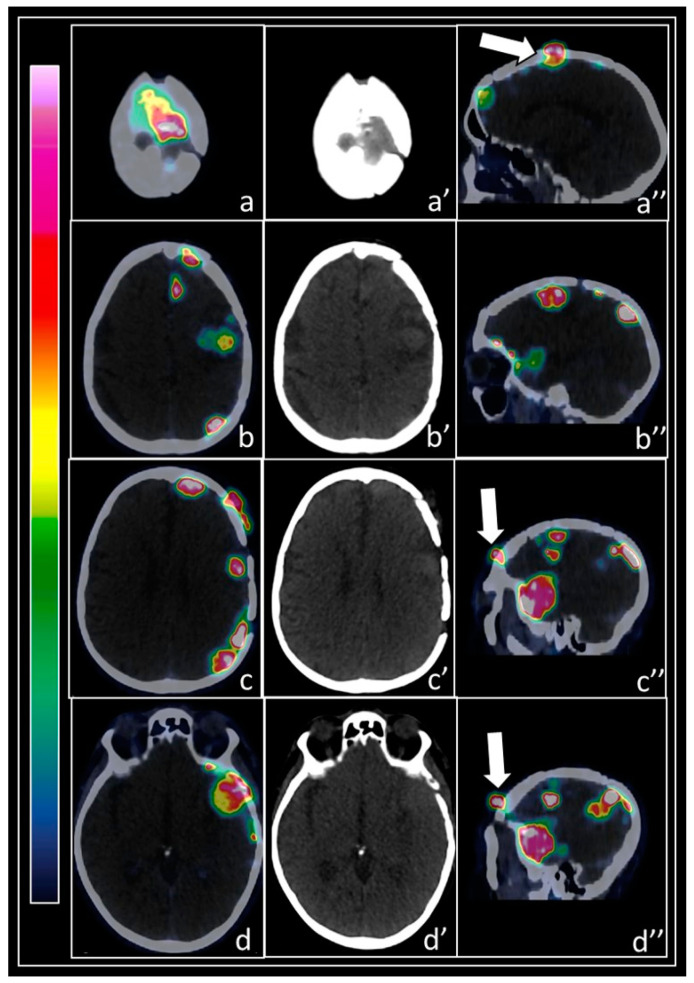
A 70-year-old patient with multiple meningiomatosis, already submitted to several consecutive interventions of surgical excision in the left-brain hemisphere and two treatments with cyberknife stereotactic radiosurgery for recurrences, was examined by brain PET/CT with ^68^Ga-DOTATOC for an evaluation of tumor extent as suggested by Laudicella et al. [34]. PET/CT displayed multiple foci of pathologic tracer uptake in the left-brain hemisphere, particularly in the cranial vertex, as shown in the axial PET/CT (**a**) and CT (**a’**) views, corresponding to a tracer-avid lesion protruding into the scalp, also evident in the correlative sagittal PET/CT view (**a’’**, arrow). The patient clinically presented skull deformation in the vertex of the skull. Other tracer-avid parafalcal and subcortical lesions were also detected, shown in the axial PET/CT (**b**) and CT (**b’**) views and correlative sagittal PET/CT view (**b’’**). Moreover, multiple cortical tracer-avid lesions were observed in the left-brain hemisphere, as evident in axial PET/CT (**c**) and CT (**c’**) details. Some of these lesions were protruding into the subcutaneous tissues in the left frontal region, as depicted in the sagittal PET/CT view (**c’’**, arrow). Finally, intense ^68^Ga-DOTATOC uptake was recorded in a slightly hyperdense area in the left temporal region, invading subcutaneous soft tissues in the ipsilateral scalp. This finding is summarized in the axial PET/CT (**d**) and CT (**d’**) views and sagittal PET/CT view (**d’’**, arrow).

As regards meningioma cells, SSTR2 is the predominant SSTR subtype, and it is present in 90% of meningiomas [32,33], therefore allowing interesting clinical applications for both diagnostic and therapeutic purposes in these tumors. The expression of these receptors may be delineated by PET with radiolabeled somatostatin analogs [34]. PET may also have a role in the assessment of tumor growth rate in well-differentiated meningiomas (this is important in order to select the optimal time point for treatment initiation). In fact, in a study by Sommerauer et al., the authors demonstrated a correlation between maximum standardized uptake value (SUVmax) and tumor growth rate in patients with WHO-grade-I- and II meningiomas [61]. Figure 1 depicts a case of a 70-year-old patient with multiple meningiomatosis evaluated with ^68^Ga-DOTATOC PET/CT. 

Regarding PRRT in meningiomas with radiolabeled somatostatin analogs, important results have been demonstrated in a recent meta-analysis by Mirian et al. In fact, in patients with treatment-refractory meningioma, PRRT allows disease control in 63% of patients, with promising results in one-year overall survival (88%, 71% and 52%, respectively, for WHO-I-, II- and III-grade meningioma, respectively) [35]. In a recent study, Parghane and collaborators evaluated the feasibility and effectiveness of therapy with ^177^Lu-DOTATATE in five patients with meningiomas (all these patients had also a concomitant NET). Three of five patients responded to treatment (two complete responses and one partial response), and all patients had no significant side effects, showing the feasibility of this therapeutic approach in meningiomas [36]. Furthermore, a study by Hartrampf et al. showed the feasibility and safety of PRRT with radiolabeled somatostatin analogs combined with external beam radiotherapy. Results demonstrated disease stabilization in 7 of 10 patients with advanced meningioma, and treatment was well tolerated (with no relevant side effects) in all patients [37]. 

^90^Y-DOTATOC can be used in radio-guided surgery of meningiomas. Thanks to radio-guided surgery (RGS), the neurosurgeon receives information regarding the location and extent of the lesion in real time, thus being able to evaluate the effectiveness of the resection by minimizing the amount of healthy tissue removed. This technique uses a radiopharmaceutical administered to the patient, which is preferentially taken from the tumor lesion in order to discriminate cancer from healthy tissue. [62]

The importance of RGS in tumor surgery of PBTs is highlighted by the fact that presurgical imaging is poorly effective in finding residues, as the operative field substantially changes during brain tumor resection. These considerations make ^90^Y-DOTATOC an optimal candidate tracer, as this technique takes advantage of the fact that ^90^Y-DOTATOC is absorbed mostly by the tumor than by healthy tissues. The patient, while waiting for surgery, is injected with ^90^Y-DOTATOC several hours in advance (considering the half-lives of the radionuclides). After removing the bulk of the tumor, the surgeon explores the site with a β^−^ probe to verify the completeness of the resection. The probe responds to the presence of β^−^ radiation with a signal, the rate of which depends on the presence of radionuclides, their spatial distribution and the probe characteristics [62].

As regards pediatric PBTs, medulloblastoma and supratentorial primitive neuroectodermal tumors (malignancies that typically affect the pediatric age) may express these receptors [53,63]. In particular, medulloblastoma cells have a high expression of SSTR2 [44]. SSTR2 expression in these types of tumor opened the door to somatostatin receptor imaging in these cases, as demonstrated in a previous study by Muller and collaborators, in which the authors assessed the usefulness of SRI in pediatric patients with medulloblastoma [64]. These important results in the field of SRI were confirmed by Fruhwald et al. The authors assessed the expression of SSTR in 13 children with malignant PBTs. SRI with ^111^In-pentetreotide SPECT was positive in seven supratentorial primitive neuroectodermal tumors and one medulloblastoma [65]. Moreover, it is important to mention the study by Abongwa et al., in which one medulloblastoma and one supratentorial primitive neuroectodermal tumor showed high uptake of ^68^Ga-DOTATOC [66]. 

The aforementioned studies demonstrated that somatostatin receptor-positive PBTs may also be treated with radiolabeled somatostatin analogs. In this context, the feasibility of therapy with ^90^Y-DOTATOC was reported by Menda et al. in children with medulloblastoma and anaplastic astrocytoma [45]. Furthermore, a case of a child with an advanced SSTR-positive medulloblastoma has been recently presented by Pizzoferro et al. The authors demonstrated the promising role of 177Lu-PRRT in last-line treatment in the case of SSTR-positive medulloblastoma. As pointed out by the biokinetic assessments, kidney radiation exposure may represent the main limitation of this treatment [46].

## 3. Radiopharmaceuticals for Neurokinin Type-1 Receptor

As regards PRRT in PBTs, an important target for radionuclide therapy is represented by neurokinin type-1 receptor, which is well expressed in these tumors, especially in gliomas [22,67]. Substance P and its precursors neurokinin A and B (peptides that belong to the family of tachykinins) are the major ligands of neurokinin type-1 receptor, a transmembrane receptor present in many cell types, such as white blood cells, fibroblasts, neurons and endothelial cells [68,69]. After ligand binding, these receptors are internalized in the cell [70]. Substance P may be easily radiolabeled using chelators such as DOTA or (1,4,7,10-tetraazacyclododecane-1-glutaric acid-4,7,10-triacetic acid (DOTAGA) [8], thus developing radiopharmaceuticals for imaging and therapeutic purposes. For therapy with radiolabeled substance P in PBTs, as mentioned below, the most reported radionuclides in the literature are ^90^Y (β-radiation-emitting radionuclide) and ^213^Bi (α-radiation-emitting radionuclide). This PRRT requires intratumoral injections of the radiopharmaceuticals [17].

Several authors evaluated the effectiveness of radionuclide therapy with radiolabeled substance P in PBTs. As early as 2007, Kneifel and collaborators demonstrated the feasibility of a protocol for radionuclide therapy with 90Y-DOTAGA-substance P in patients with malignant gliomas (in this study were included four patients with glioblastomas, two with anaplastic gliomas and six with low-grade gliomas). In these subjects, pretherapeutic SPECT scans were performed after the injection of ^111^In-DOTAGA-substance P. These scintigraphic scans predicted the intratumoral localization of dose deposition with precision, suggesting the feasibility of this approach [71].

Cordier et al. reported the efficacy of neoadjuvant radionuclide therapy with ^90^Y-DOTAGA-substance P (before surgical resection) in patients with glioblastoma, showing its feasibility as well. Administration of the radiopharmaceutical was performed through intratumoral injections. The results of this phase I study were relevant in terms of low toxicity and prognosis. In fact, no relevant side effects were documented (in some cases only an increase in perifocal edema) and neoadjuvant therapy with ^90^Y-DOTAGA-substance P allowed a higher extent of surgical resection of glioblastomas [22,26]. 

As regards radionuclide therapy in PBTs and neurokinin type-1 receptor, few studies evaluated the safety and the effectiveness of therapy with α-radiation-emitting radionuclide ^213^Bi. It is important to underline that in comparison with the β-radiation-emitting radionuclide ^90^Y, α-radiation of ^213^Bi shows reduced mean tissue penetration (81 μm vs. 5 mm) [17], therefore showing very low toxicity [22]. In 2010, Cordier and collaborators assessed the feasibility of radiotherapy with local injections of ^213^Bi-DOTA-substance P in five patients with critically located gliomas (two glioblastomas, one WHO-grade-III glioma, two WHO-grade-II gliomas). PRRT with intratumoral injections of ^213^Bi-DOTA-substance P was safe and well tolerated by these patients, without relevant side effects. In addition, authors suggested that PRRT with α-emitters radionuclides in PBTs allows a similar efficacy compared to radiotherapy with β-radiation-emitting radionuclides, with lower toxicity and consequently reduced damage to adjacent lesion areas (in this regard, it is important to mention the shorter half-life of ^213^Bi in comparison to ^90^Y, respectively, 46 min vs. 2.7 days) [72,73].

This promising therapeutic approach with ^213^Bi-DOTA-substance P was confirmed by Królicki et al. in 2018. Twenty patients with recurrent glioblastoma multiforme were treated with ^213^Bi-DOTA-substance P, and injections of the tracer were performed through intracavity or intratumoral injections in 2-month intervals. The results of this study demonstrated the safety of this radionuclide therapy. Treatment was well tolerated in all patients, and no severe side effects occurred. Moreover, the median overall survival from the diagnosis of recurrence was 10.9 months, showing comparable results with other treatment options. Therefore, the authors demonstrated that radionuclide therapy with ^213^Bi-DOTA-substance P is a valid alternative therapeutic approach in patients with recurrent glioblastoma multiforme [27]. The same research group demonstrated the safety and feasibility of therapy with ^213^Bi-DOTA-substance P in patients with glioblastoma multiforme in another interesting study as well, in which Królicki et al. reported the results of this therapy in nine subjects. Treatment was well tolerated by all patients (only mild adverse reactions were reported, such as headaches due to transient edema), and the median overall survival after the beginning of therapy with ^213^Bi-DOTA-substance P was 16.4 months [28], underling the promising role of this treatment as a reliable alternative to conventional therapies. 

^225^Ac is another well-known α-radiation-emitting radionuclide, with a longer half-life in comparison to ^213^Bi (9.9 days vs. 46 min) [74]. Krolicki and collaborators demonstrated the safety and feasibility of radionuclide therapy with ^225^Ac-DOTA-substance P in a group of 21 patients with recurrent glioma (1 patient with WHO-grade-II glioma, 8 patients with WHO-grade-III glioma, 12 patients with WHO-grade-IV glioma). Moreover, these patients underwent a PET/CT scan with ^68^Ga-DOTA-Substance P (coinjected with ^225^Ac-DOTA-substance P) in order to assess the biodistribution. Treatment with ^225^Ac-DOTA-substance P was well tolerated, showing promising results in terms of overall survival (28–62 weeks from the diagnosis of recurrence) [29].

## 4. Prostate-Specific Membrane Antigen (PSMA)-Targeted Radiopharmaceuticals

PSMA is a transmembrane glycoprotein highly expressed in prostate cancer, and several PSMA-targeted radiopharmaceuticals have been developed for imaging (PET and SPECT) and treatment of prostate cancer [75,76,77]. PSMA is encoded by the gene FOLH1 mapped on chromosome 11, and it is involved in enzymatic peptidase activities and activation of signaling pathways [78].

Despite the term “specific”, a high expression of this protein may be found in other benign or malignant conditions such as inflammations or nonprostatic tumor-associated neovasculature [78]. Regarding the last point, several immunochemistry studies have demonstrated the presence of PSMA in the endothelial cells of tumoral neovasculature [79], and it is well known that several malignancies may express high levels of PSMA, such as breast cancer [80], melanoma [81], non-small-cell lung cancer [82] or lymphoma [83]. 

This glycoprotein has an internalization fragment that can internalize therapeutic molecules into the cell, so PSMA is involved in integrin signaling in vitro and angiogenesis in vivo by the generation of proteolysis of matrix protein laminin that activates endothelial cells [84]. These results suggest a role of PSMA in tumor vasculature and that inhibition of PSMA could lead to new antiangiogenic therapies. PSMA expression has been demonstrated in endothelial cells and deals with neovascularization, while only a small part is represented in tumor cells or cancer-free brain. According to this, PSMA PET could be useful for antiangiogenic therapies or PSMA-targeted drugs. The delivery and retention of PSMA particles to tumors depend on two factors: high tumor perfusion and PSMA expression in the tumor epithelium [85]. Tumor angiogenesis has increasing value as a marker in diagnosis and as a therapeutic target of brain tumors as well. According to this, it was shown that in some tumors, PSMA is widely expressed in neovascularization, with minimum or nonexpression in tumor cells or normal endothelium. PSMA was found to be upregulated in the blood–brain tumor barrier in metastatic lesions by breast cancer, with poor expression in normal blood–brain barrier [86]. Normal blood–brain barrier and blood–brain barrier in metastatic lesions have many differences, such as few adhesions of endothelial cells that make it more permeable, even if does not allow a significant response to therapy because the concentration of drugs in metastatic brain lesions is lower than that of systemic metastasis [86].

As regards PBTs, it was shown that patients with a high/increasing level of PSMA in neovasculature in tumoral recurrence (glioblastoma multiforme) survived shorter than with low/decreasing vascular expression, so this seems to be a negative prognostic marker. Thus, PSMA expression in this malignant brain tumor might present a target for theragnostic approaches above all in recurrence [87]. 

Several recent studies with PET have demonstrated the high expression of PSMA in the neovasculature of PBTs. Matsuda et al. used ^89^Zr-Df-IAB2M (a radiolabeled anti-PSMA antibody) for the evaluation of brain tumor neovasculature and the possibility of detecting PSMA expression using PET. Final results showed that PSMA was highly expressed in high-grade gliomas, with no expression in radiant necrosis tissue visualized by PET with ^89^Zr-Df-IAB2M. These results suggested also a potential value in the prediction of treatment efficacy in glioma and differential diagnosis with radiation necrosis. PSMA in endothelium was found in 97% of glioblastomas (41 patients were analyzed) and 75% of astrocytomas (4 patients were involved in the study). In these cases, PET with ^89^Zr-Df-IAB2M showed high uptake of the radiolabeled PSMA antibodies in brain tumors [88]. In 2017, Sasikumar et al. demonstrated the feasibility of ^68^Ga-PSMA11 PET/CT for imaging of PBTs and its superiority in comparison with ^18^F-fluorodeoxyglucose (^18^F-FDG) PET/CT. Ten patients with PBTs were enrolled in the study. In all cases (including glioblastoma multiforme, other gliomas and atypical meningiomas), lesions showed high uptake of ^68^Ga-PSMA11. In addition, ^68^Ga-PSMA11 PET/CT allows better visualization of the tumor compared to ^18^F-FDG PET/CT [89]. In 2019, Verma and collaborators evaluated 10 patients with PBTs using ^68^Ga-PSMA-HBED-CC (^68^Ga-PSMA 11) PET/CT. The authors demonstrated that high-grade gliomas express higher PSMA expression in comparison to low-grade gliomas, with an average SUVmax of 16.93 in high-grade gliomas and 2.93 in low-grade gliomas [23]. 

The aforementioned studies clearly demonstrate the high expression of PSMA in PBTs (especially in high-grade gliomas) and, consequently, open the door to attractive theragnostic and therapeutic approaches in these tumors, adding new potential weapons to conventional treatments. Despite this evidence, to the best of our knowledge, only one case is reported in the literature regarding PRRT with PSMA-targeted radiopharmaceuticals in PBTs. This case regarded a 37-year-old man with a recurrent glioblastoma multiforme. Magnetic resonance imaging (MRI) and ^68^Ga-PSMA PET showed the recurrence, and because PSMA expression was demonstrated, the patient began treatment with ^177^Lu-PSMA 617. The lesion showed a significant reduction after three cycles of therapy with ^177^Lu-PSMA 617, with an improvement in clinical conditions and symptoms as well [30]. Further studies are needed in order to confirm the value of PRRT with PSMA-targeted radiopharmaceuticals in PBTs, but this initial evidence is promising. Moreover, we should underline the favorable characteristics of PSMA 617 that make it “ideal” for theragnostic purposes in patients with PBTs: favorable tumor uptake, rapid clearance through the kidneys and extended tumor retention [90]. For the optimal features described, this peptide was also utilized for the treatment of other intracranial lesions such as brain metastases, especially in patients with prostate cancer [91]. 

## 5. Discussion

As reported before, conventional treatments of PBTs include surgery, chemotherapy and radiotherapy, usually combined. In several cases, treatment is complicated and ineffective due to several factors (for example, due to the blood–brain barrier or the localization of tumoral lesion), and prognosis of patients with PBTs is poor. In addition, it is important to highlight the possible complications of conventional treatments. For example, as regards postoperative complications, thromboembolism (60% of cases after surgery in patients with gliomas) and seizures (30% of patients after surgery) are common [7]. Side effects and toxicity of some chemotherapy drugs are frequent, such as weight loss, fatigue and neuropathy, which affect the health-related quality of life of patients with PBTs [92]. Moreover, radiation toxicity due to conventional radiotherapy for brain lesions is well known and reported in several studies [93].

New safety and feasible therapeutic strategies for patients with PBTs are strongly needed. As we have highlighted in the studies and cases reported in this review, PRRT may have a promising and attractive role in this context. In addition, radiolabeled peptides may allow a theragnostic and personalized approach, evaluating potential targets (such as somatostatin receptors, neurokinin type-1 receptor or PSMA) and predicting with SPECT or PET imaging if a patient will benefit from PRRT. It is also important to mention that the development of theragnostic radiopharmaceuticals and agents is growing, with the utilization of these agents in several malignancies reported in the literature [1]. 

As regards PRRT with radiolabeled somatostatin analogs, the feasibility of this therapy in PBTs has been demonstrated in patients with medulloblastoma, high- and low-grade glioma, meningioma [1,94] and pituitary tumors [95]. As we have highlighted in the studies reported in this review, a pretherapeutic assessment of the expression of SSTR2 in these patients is necessary in order to select cases with a high chance of benefit from PRRT with radiolabeled somatostatin analogs. Meningioma expresses high levels of SSTR2 [32], allowing the use of radiolabeled somatostatin analogs for imaging and therapeutic purposes. It is important to mention that the European Association of Neuro-Oncology (EANO) considered these approaches as important “future directions” regarding the clinical management of treatment-refractory meningioma [96]. 

In the case of lesions with low expression of SSTR, alternative strategies and specific methods to deliver the radiopharmaceutical to PBTs have been suggested. In this regard, it is important to underline that intraarterial administration of the radiolabeled somatostatin analog may increase the efficacy of PRRT in meningiomas with low levels of SSTR2, as suggested in an interesting report by Braat and collaborators [16]. Moreover, in comparison to intravenous administration, intraarterial administration of the radiopharmaceutical led to an increased tumor uptake, as demonstrated in a recent study by Vonken et al., who evaluated patients with meningiomas refractory to surgery and radiotherapy [97]. Intraarterial administration of the radiopharmaceutical has been demonstrated to be useful in PBTs with other therapeutic agents such as chemotherapeutic drugs as well, increasing the intratumoral drug concentration [98]. Further studies are needed, but this approach may be useful and effective in meningiomas (and other PBTs) using radiolabeled somatostatin analogs. 

Patients affected by other types of PBTs such as pituitary tumors may benefit from PRRT with radiolabeled somatostatin analogs, especially GH and TSH tumors (as we showed, these tumors express high levels of SSTR2). It is important to point out that gonadotroph tumors (one-third of anterior pituitary tumors) usually express higher levels of SSTR3 rather than SSTR2. In this case, DOTATOC may be preferred over DOTATATE for diagnostic and therapeutic purposes (DOTATOC presents higher affinity for SSTR3 in comparison to DOTATATE) [58,99]. As regards pretherapeutic assessment of SSTR in pituitary tumors, important results may be obtained from the results of ongoing clinical trials. Worthy of note is the possible correlation between SUVmax in ^68^Ga-DOTATOC PET and SSTR expression of pituitary tumors (https://clinicaltrials.gov/ct2/show/NCT02419664?term=somatostatin&cond=Brain+Tumors&draw=2&rank=8).

Neurokinin type-1 is an attractive target for PRRT in PBTs. As we demonstrated, there is a lot of evidence regarding the possible utilization of radiolabeled substance P for therapeutic purposes in gliomas, demonstrating safety and feasibility. As we showed, the low toxicity of this PRRT is mainly related to transient edema after treatment and the possible damage to adjacent tumor areas. In this regard, there are studies that suggested the possible use of α-emitting radionuclides (^213^Bi or ^225^Ac). In fact, the very short range of α-radiation (less than 0.1 mm) [100] may be a considerable advantage in comparison to therapy with β^−^-emitting radionuclides, killing selectively cancer cells and sparing healthy tissues and organs. As regards α-therapy, O6-methylguanine-DNA-methyltransferase (MGMT) promoter methylation status does not affect the effect of radiation, thus overcoming resistance to chemotherapy with alkylating agents [74]. Moreover, it is important to underline that the choice of appropriate radionuclides is crucial, and it depends on the size of the lesion. For larger tumors, a radionuclide with a long half-life such as ^225^Ac (half-life: 9.9 days) may be preferred, as it allows a better distribution of the radiopharmaceutical in the lesion. On the other hand, radionuclides with a short half-life such as ^213^Bi (half-life: 46 min) may be more useful for small brain lesions [29]. 

PRRT and theragnostic applications with PSMA-targeted radiopharmaceuticals, in particular with ^177^Lu-PSMA 617, are experiencing growing interest in oncology, especially for the management of patients with castration-resistant prostate cancer [101]. PSMA is also a marker of neovasculature in several other malignancies. As regards PBTs, neovasculature is an important component of tumor biology in high-grade gliomas, especially in the case of glioblastoma multiforme. In this context, new antiangiogenetic therapies have been developed [102]. Patients affected by highly vascularized PBTs such as glioblastoma multiforme [103] may benefit from PRRT with ^177^Lu-PSMA 617. To the best of our knowledge, there is only one report in the literature regarding the utilization of ^177^Lu-PSMA 617 in a patient with glioblastoma multiforme, and further large-scale studies are needed. However, this therapeutic approach seems to be promising. In addition, PSMA 617 may be radiolabeled with ^225^Ac [104,105], opening the door to possible α-therapy with PSMA-targeted radiopharmaceuticals. It is also important to mention the possible toxicity of radiolabeled PSMA-targeted radiopharmaceuticals, as demonstrated in a pilot study by Khreish and collaborators, evaluating patients with prostate cancer in treatment with ^177^Lu-PSMA 617 and ^225^Ac-PSMA 617. Haematotoxicity and xerostomia were the most common side effects reported by the patients [105].

As concerns promising approaches with other radiopharmaceuticals, it is important to underline the possible targeting of gastrin-releasing peptide receptor (GRPR). GRPR is a bombesin family receptor expressed in several tumors and especially in optic-pathway glioma [1]. Optical pathway glioma is most frequent in children and is a rare astrocytic tumor (3–5% of all pediatric intracranial tumors) [106]. As concerns treatment, surgery is usually complicated, and the main options are often radiotherapy and chemotherapy [1]. In this context, it is very important to highlight the possible role of GRPR-targeted therapy in these tumors, as underlined in an interesting study by Zhang et al. In fact, the authors showed the feasibility of ^68^Ga-NOTA-AcaBBN(7–14) PET in eight children with optical pathway glioma, and all lesions showed high uptake of the radiopharmaceutical [107]. To the best of our knowledge, there are no reports in the literature concerning these tumors and GRPR-targeted radionuclide therapy, but the study by Zhang and collaborators opens the door to possible and attractive theragnostic approaches.

## 6. Conclusions

The treatment of PBTs is usually complicated, and considering the poor prognosis in several cases, new weapons and strategies are strongly needed. This review underlined the potential contribution of PRRT and possible perspectives in this field. As demonstrated, PBTs such as gliomas, meningiomas, pituitary tumors and medulloblastomas may benefit from PRRT. Taken together with data from the literature, PRRT seems to be feasible and reliable in the treatment of PBTs. Moreover, PRRT also allows interesting theragnostic approaches with the selection of potential patients, thus permitting a personalized therapy. Further studies on larger samples are needed to determine the overall efficacy of PRRT in PBTs, but the growing and groundbreaking developments in the field of theragnostics may facilitate this approach. 

## Figures and Tables

**Table 1:** Primary brain tumors and peptide receptor radionuclide therapy (PRRT): possible applications.

Primary Brain Tumors	Median Survival	Possible Target for PRRT	Comments
**Gliomas**	Low-grade gliomas: 7.3 years [18]. High-grade gliomas: 18 months [18]; glioblastoma multiforme is the most lethal form (median survival: 10–15 months) [4,5,6].	Somatostatin receptor subtype 2 (SSTR2): present in 25% of gliomas [19]. Variable expression of SSTR2 in low- and high-grade gliomas [19,20,21]. Neurokinin type1 receptor: well expressed in low and high gliomas [22]. Prostate-specific membrane antigen (PSMA): higher expression in high-grade gliomas in comparison to low-grade gliomas [23].	PRRT with radiolabeled somatostatin analogs: pretherapeutic assessment of SSTR2 expression is needed [24]. In somatostatin receptor-positive gliomas, PRRT seems to be safe and feasible [24,25]. Effectiveness of PRRT with radiolabeled substance P (using α- and β^−^-radiation–emitting radionuclides) demonstrated in low- and high-grade gliomas [22,26,27,28,29]. Only one case is reported in the literature regarding PRRT with PSMA-targeted radiopharmaceuticals in a patient with glioblastoma multiforme [30].
**Meningiomas**	Survival decreased with grade. Median survival of the most aggressive form (WHO grade III, also called malignant meningioma) is 4.1 years [31].	Meningioma expresses high levels of SSTR2 [32]: 90% of meningiomas express SSTR2 [33].	The expression of SSTR2 receptors may be delineated by positron emission tomography (PET) with radiolabeled somatostatin analogs [34]. Important results of PRRT with radiolabeled somatostatin analogs have been demonstrated [35,36], even in association with external beam radiotherapy [37].
**Pituitary Tumors**	8 months for the most aggressive form (pituitary carcinoma) [38].	Pituitary tumors may present SSTR, with a heterogeneous expression [39]: growth hormone (GH)- secreting tumors and thyroid-stimulating hormone (TSH)-secreting tumors mainly express SSTR2 and SSTR5; prolactinomas mainly express SSTR1 and SSTR 5; nonfunctioning and gonadotroph tumors mainly express SSTR3; adrenocorticotropic (ACTH)-secreting tumors mainly express SSTR5.	Only few reports on the possible applications of PRRT with radiolabeled somatostatin analogs [40,41]. Somatostatin receptor imaging has a potential central role in the selection of potential patients [42]
**Medulloblastomas**	80 months [43].	Medulloblastoma cells have high expression of SSTR2 [44].	Promising results of PRRT with radiolabeled somatostatin analogs in somatostatin receptor-positive medulloblastoma [45,46].

**Table 2:** Physical characteristic of radionuclides used for peptide receptor radionuclide therapy (PRRT) in primary brain tumors (PBTs).

Radionuclide	Emission	Half-Life	Maximum Energy (Emax)	Range Max of Radiation in Tissue
**^90^Y**	Beta minus	64 h	2.27 MeV	11 mm
**^177^Lu**	Beta minus	6.6 days	0.49 MeV	2 mm
**^213^Bi**	Alpha	46 min	8.4 MeV	40–80 µm
**^225^Ac**	Alpha	9.9 days	8.4 MeV	85 µm

## Data Availability

Data are within the article.
